# Obesity and Sleep-Related Breathing Disorders in Middle East and UAE

**DOI:** 10.1155/2016/9673054

**Published:** 2016-12-01

**Authors:** Mayank G. Vats, Bassam H. Mahboub, Hassan Al Hariri, Ashraf Al Zaabi, Deepa Vats

**Affiliations:** ^1^Department of Respiratory and Sleep Medicine, Rashid Hospital, Dubai, UAE; ^2^College of Medicine, University of Sharjah, Sharjah, UAE; ^3^Department of Respiratory and Sleep Medicine, Sheikh Zayed Military Hospital, Abu Dhabi, UAE; ^4^Pediatric ENT, Al Jalila Children's Hospital, Dubai, UAE

## Abstract

A pandemic of obesity is sweeping all across the globe and the Middle East region also does not remain untouched by this prevailing pandemic. In fact, as per WHO report, Kuwait has the second highest obesity prevalence followed closely by other Middle East (ME) countries, namely, Qatar, Saudi Arabia, and United Arab Emirates (UAE). Apart from direct medical, psychological, and quality of life related adverse effects of obesity, many indirect medical comorbidities, namely, obstructive sleep apnea (OSA), obesity hypoventilation syndrome (OHS), diabetes mellitus (DM), hypertension (HTN), and metabolic syndrome, imposes a significant health burden on the individual and community with consequent morbidity and mortality. The purpose of this review is to shed light on the very high prevalence of obesity, undiagnosed sleep apnea, and other obesity related disorders with discussion of the contributing factors specific to the region including the fair insight into the current status of sleep medicine services in Middle East and UAE despite huge number of patients having undiagnosed sleep disorders. We will also suggest to control this epidemic of obesity and OSA so that the corrective measure could be taken at health ministry level to help people of this region to fight against obesity and related disorders, primarily OSA.

## 1. Introduction

Obstructive sleep apnea hypopnea syndrome (OSAHS) and sleep-related breathing disorders (SRBD) represent a significant paradox in medicine because they have been recognized only in recent decades and are still regarded as a niche interest by most of the clinicians (other than respiratory, neurology, and otolaryngology specialists).

On the other hand, the OSAHS syndrome is common and current epidemiological data indicate that OSAHS is second only to asthma in the prevalence league table of chronic respiratory disorders. There have been a considerable number of studies that have addressed the prevalence of OSAHS varying from 0.5% to 19 depending upon the criteria used to define the OSAHS and the population studied [1, 2].

The average prevalence of OSA is around 4–6% for middle age men and women; however, all these studies are from the western world; as such, no comprehensive data is available on the prevalence of OSAHS in the Middle East, UAE, and other countries primarily owing to the lack of awareness among clinicians and community, scarcity of resources, and other cultural and social beliefs.

## 2. Obesity in Middle East and UAE

The Middle East region, including the Arabian Peninsula, Eastern Mediterranean, Turkey and Iran, and North Africa, is no exception to the worldwide increase in obesity and subsequent medical comorbidities. Subsequently, this trend has been named as “New World Syndrome” [3]. Discovery of oil with subsequent increase in wealth led to lifestyle changes which is the most important contributing factor giving rise to high incidence of obesity.

An epidemic of obesity is sweeping across the Middle East with no good signs of control and in many surveys it has been confirmed and projected that the incidence and prevalence of obesity and OSAHS will increase over the next few decades considering the high prevalence of obesity and a very complex interrelationship between obesity and OSAHS.

Prevalence of obesity and consequent sleep-related breathing disorders are increasing rapidly in the Middle East region (UAE, Kingdom of Saudi Arabia (KSA), Qatar, Bahrain, Kuwait, and Oman) with contribution from sedentary/westernized lifestyles, fast food (rich in fat, salt, sugar, and refined starches), adverse outdoor weather conditions, genetics, and complex interplay of many other factors. Considering the high prevalence of obesity and OSAHS, we can no longer ignore the direct and indirect cost of obesity in terms of consequent health problems, healthcare cost, and morbidity and mortality.

## 3. High Prevalence of Obesity

The high prevalence of obesity could be a direct effect of the high economic class and lifestyle changes in Gulf countries over the past few decades.

A recent study published by the London School of Hygiene and Tropical Medicine revealed that Kuwait is the second most obese nation in the world lagging behind the US (WHO data) [4]. Kuwait ranked the fourth most obese country on the planet according to a report published in medical journal “The Lancet.” More than 50% of women in Kuwait are overweight or obese (≥50% of population are overweight; ≥10% are morbidly obese), and in KSA, ≥35% of the population are clinically overweight followed by Libya, Qatar, and Samoa, that are considered obese according to the Global Burden of Disease Study [5].

In KSA, prevalence of obesity in adults (age: 30–60 years) has increased by 1.5% for women and 4.1% for men every year [6]. A study conducted at King Khalid University and King Fahd National Guard primary health clinics (PHC) in Riyadh found that around one-third of middle-aged Saudi male participants showed symptoms of sleep apnea [7].

In Qatar and Kuwait, 35% and 36% of male and 45% and 48% of female adults were found to be obese, respectively [8]. Equally alarming explosion of obesity has been observed in young people in other Middle East countries.

Forbes magazine ranked UAE to be number 18 on the list of the world's fattest countries, estimating 68.3% of its citizens to be overweight making UAE one of the top countries having high prevalence of obesity [9].

According to the latest available study by the WHO, 67% of Emirati men and 72% of Emirati women are overweight and around 39.9% of UAE women and 25.6% of men are obese (the 7th and 9th highest proportion in the world, resp.) [10].

Ministry of Health, UAE, carried out study on national nutrition which revealed that approximately 33% of married women were overweight and 38% were obese. Among married men, 40.3% were overweight but only 15.8% were obese [4]. In general, 20% of population suffers from obesity, which is more than the obesity rates in USA.

At an obesity conference in Abu Dhabi, in 2010, it was revealed that 71% of the Emirati adult population is obese, while in a recent survey in Abu Dhabi, 35% of the population was classified as obese and 32% as overweight [10].

Overweight and obesity therefore have risen dramatically to alarming heights in the Middle East and UAE over the past decade and became a major health problem (inviting other chronic diseases such as HTN, DM, and SRBD) attributed primarily to urbanization and the sedentary modem lifestyle. Studies have also found that expatriate workers are far more likely to suffer from obesity after spending a period in the UAE due to the sedentary life/outside climate and dietary habits.

A wellness survey in UAE (*n* = 700 lay individuals) revealed that 38% of participants declared themselves as overweight or obese; 82% of these obese participants told surveyors that overweight people are unhealthy and overweight negatively affects a person's day-to-day activities. However, nothing was done to reduce the weight by these participants reflecting poor education/time restrictions/low motivation and other undefined factors. More than 72% of participating healthcare professionals (HCP) (*n* = 50) believed that the main cause of obesity in the UAE is sedentary lifestyle with lack of awareness about the consequences of obesity. This was followed by westernized high caloric diet, cultural/genetic predisposition, and adverse climate. This high prevalence of obesity is the stark cost of the sedentary lifestyle both Emiratis and expatriates living here, along with the addiction to western style fast food [11].

Recent study in Dubai on prevalence of OSA in UAE (*n* = 1200 participants attending PHC clinics) revealed that almost 24% of males and 21% of females in the UAE probably have sleep disorders primarily related to OSAHS [12].

## 4. Consequences of Obesity and OSA

Middle East region is bearing the consequences of obesity and OSA with the ever-increasing incidence and prevalence of noncommunicable diseases, namely.

### 4.1. Diabetes Explosion

A key factor driving healthcare costs in Middle East and UAE is growing incidences of lifestyle-related health problems such as DM and cardiovascular diseases, often seen as a direct consequence of obesity and OSA. The burden of DM is expected to surge over the next decades in the Middle East with expected 60 million diabetics in year 2030. UAE has high obesity prevalence and the 2nd highest rate of diabetes in the world. According to the International Diabetes Federation (IDF), around 34.6 million people in the Gulf cooperative countries (GCC) (9.2% of the adult) have DM and this number is expected to almost double to 67.9 million by 2035 [13]. The Health Authority-Abu Dhabi (HAAD) expects healthcare costs for UAE nationals to rise fourfold by 2030. Indeed, among emerging markets, the UAE and Qatar spend the highest diabetes-related healthcare expenditures.

Worryingly, in the Middle East region, the prevalence of DM among younger age groups is substantially higher than the global average. KSA has the highest number of children with type 1 DM in GCC region. Weight reduction, medical nutrition therapy, and therapeutic lifestyle can halve the risk of DM and obesity; however, these methods have never been employed by majority of obese persons.

### 4.2. Cardiovascular Disease

 Cardiovascular diseases are also on steep surge as a consequence of obesity and OSA in Middle East region and expected to continue on rise over the coming decades with significant morbidity and mortality. Around 70% of the deaths in UAE are caused by cardiovascular disease with significant contribution from obesity and OSA.

### 4.3. Road Traffic Accidents

Studies show that up to 20% of road traffic accidents are sleep-related with over 1000 road traffic deaths a year in UAE, and over 6300 road traffic deaths in Saudi Arabia [2007 police data] [14]. They recommended that a well-designed sleep testing and treatment program in collaboration with road traffic authority could reduce road traffic fatalities significantly.

### 4.4. Economic Burden

All Middle East countries have seen huge hikes in medical budgets over the last few years. In 2011, GCC spent around $28.9 billion on healthcare. It is estimated that this figure will be $44 billion by 2014 and 60 billion USD or more by 2025 [13].

We do not have the exact cost for the UAE but, with the country ranking high globally in obesity and diabetes prevalence, the economic impact could run into billions and it is the “*need of the hour*” to estimate the cost of obesity and related health problems to take steps to curb the healthcare cost.

## 5. Prevalence of OSAHS in the Middle East and UAE

One recent study conducted in PHC setting in Dubai with the goals of estimating the prevalence of symptoms and risk of OSAHS and the relationship between obesity and OSAHS found that 20.9% of patients who attended PHC were at high risk for OSAHS (22.9% males, 19.5% females) [15].

Similar type of study from KSA revealed that 33.3% of patients were considered as high-risk patients for OSA in PHC setting; hence, 1 out of 3 middle-aged Saudi males is at risk for OSA and 39% of females were considered as high-risk patients for OSA [16, 17].

In Jordan, the overall risk for OSA was 16.8% (19.3% men versus 14.7% women, resp., *p* < 0.04) [18]. In a general population surveys in Iran for investigating people at risk of OSA (using Berlin questionnaire), the surveyors found that 27.3% of subjects (men 19%; women 8.3%) were at high risk for OSA [19].

One UAE study with the objectives of assessing the prevalence of obesity hypoventilation syndrome (OHS) among OSA patients revealed that 8.5% [5.4–13.1%; CI 95%] of the studied patients had OHS [20].

## 6. Sleep Services in the Middle East and UAE

Despite extensive internet search to find out the status of sleep medicine services in Middle East countries, we could not find many details due to scarcity or nonpublished data. Some of the evidence could be gathered from few Middle East countries; considering the same socioeconomic background and ethical and medical issues, we can assume that the sleep medicine services status must be the same or worse in most of these countries where no data could be found.

A national survey conducted in KSA in 2007 quantitatively assessing sleep medicine service revealed that sleep medicine services were underdeveloped in the KSA compared to developed countries. The survey identified nine sleep disorders facilities; seven were defined as sleep disorders centers that provide clinical diagnostic and therapeutic services for patients with different sleep disorders, and two were defined as sleep laboratories that provide diagnostic and therapeutic services limited to sleep-related breathing disorders such as OSA. Only two hospitals reported having pediatric sleep medicine specialists, and four facilities reported having the required setup to perform sleep studies for children. Administratively, all surveyed sleep disorders facilities are under pulmonary medicine services [21].

The per capita polysomnography rate in the KSA was 7.1 per year per 100,000 people, compared to 18.3–427 in developed countries. The number of beds designated for sleep studies per 100,000 people was 0.06 in the KSA compared to 0.3–1.5 in developed countries. Despite the limited number of beds for sleep studies, the overall occupancy rate was 45.7% (61.1% in government hospitals versus 18.0% in private hospitals) [21]. Possible explanations for the low occupancy rate include an inadequate number of trained sleep technologists and the fact that most insurance companies do not cover the cost of polysomnography in the KSA.

In another national survey done in 2013 with the aim to find out the status of sleep medicine services in KSA, 18 sleep facilities in KSA were identified. The estimated per capita number of beds/year/100,000 people was 0.11 and the per capita polysomnography (PSG) rate was 18.0 PSG/year/100,000 people [22]. Improvement in the sleep medicine facilities in KSA between these two national surveys (conducted 6 years apart) reflects the increasing incidence and prevalence of obesity, OSA, and the increased awareness among physicians and general population about sleep disorders.

## 7. Current Status of Sleep Medicine Services and the Problems Encountered in UAE

Sleep Medicine Services in UAE are still in neonatal stage; considering the population prevalence of OSA more than 7–10% in UAE, we need many more dedicated sleep labs and trained physicians and technicians. In UAE, currently 8 specialized sleep labs (1 bed each) are functioning that too only in tertiary level hospitals and are managed by certified sleep physicians; however, the number of qualified sleep technicians is few (most of the sleep studies are done by respiratory therapists only) with approximate number of sleep tests of 1500–2000 per year. Although few companies are doing home sleep test, these companies are more inclined for selling of costly positive airway pressures (CPAP/BIPAP) machines with financial objectives and hence patients incline to get the diagnosis and treatment done in their own countries (personal experience in a large tertiary hospital in UAE).

There is very poor awareness in physicians and public about sleep aponea. Unfortunately, signs and symptoms of OSA are considered as part of life by patients and they learnt to live with it many years ago without knowing about its significance and the risks of not getting treatment.

## 8. Suggestions to the Health Authorities

Sleep medicine specialty services seem to be underdeveloped in the Middle East and UAE (cf. developed countries) due to the abovementioned factors. If we compare the prevalences of obesity and extrapolate these values in relation to the possible prevalence of OSAHS (based on applying the data from the US/western studies on prevalence of obesity and OSAHS), we would find a huge number of undiagnosed patients suffering from OSAHS and SRDB in the Middle East and UAE but unfortunately sleep medicine facilities are almost negligible and there is an urgent need to develop this specialty for the benefit of the community.

Many obstacles face the progress and development of this specialty including inadequate number of trained sleep specialists/technicians, lack of fund, poor awareness in physicians and public, and insurance coverage. A collaborative organized work involving sleep specialists, multispecialty hospitals, health authorities, and insurance companies is needed to overcome these obstacles. Additionally, local training programs are needed to train specialists/sleep technicians.

## 9. Conclusion

A holistic approach including intensive ministerial and administrative efforts should be taken in order to prevent obesity including public education and awareness emphasizing the early diagnosis and treatment of obesity, OSA, and other consequent medical problems in order to curb the numbers of these curable diseases in the Middle East and UAE can hopefully bring down the staggering rates of obesity and the associated comorbidities to ensure that the population remains healthy and prosperous forever.

Insurance companies should come forward for covering the cost of management of OSA as a “medical syndrome” (rather than considering it as simple sleep problem or misconception that this is a psychiatry problem), because there are much higher healthcare costs involved in obesity and OSA (direct and indirect); hence, insurance companies could save millions of dollars which they are spending on the treatment of HTN, heart failure and DM, and road traffic accidents (these are the consequences of untreated OSA/OSAHS), all because sleep disorders are not treated and not covered by insurance companies.

Collaborative efforts are needed between sleep specialists/technicians/social organizations to mass educate public through different media channels and to organize educational programs targeting the patients with obesity and OSA.
